# Stable and Efficient Dye-Sensitized Solar Cells and Supercapacitors Developed Using Ionic-Liquid-Doped Biopolymer Electrolytes

**DOI:** 10.3390/molecules28135099

**Published:** 2023-06-29

**Authors:** Subhrajit Konwar, Diksha Singh, Karol Strzałkowski, Mohamad Najmi Bin Masri, Muhd Zu Azhan Yahya, Markus Diantoro, Serguei V. Savilov, Pramod K. Singh

**Affiliations:** 1 Center for Solar Cells & Renewable Energy, Department of Physics, Sharda University, Greater Noida 201310, India; 2 Institute of Physics, Faculty of Physics, Astronomy and Informatics, Nicolaus Copernicus University, Grudziadzka 5, 87-100 Torun, Poland; diksha@doktorant.umk.pl (D.S.); skaroll@fizyka.umk.pl (K.S.); 3 Faculty of Bioengineering and Technology, Universiti Malaysia Kelantan, Kota Bharu 16100, Malaysia; najmi.m@umk.edu.my; 4 Faculty of Defence Science and Technology, Universiti Pertahanan Nasional Malaysia (UPNM), Kuala Lumpur 57000, Malaysia; mzay@upnm.edu.my; 5 Department of Physics, Faculty of Mathematics and Natural Science, Universitas Negeri Malang, Semarang 5, Malang 65145, Indonesia; markus.diantoro.fmipa@um.ac.id; 6 Department of Chemistry, Lomonosov Moscow State University, 1–3 Leninskiye Gory, 119991 Moscow, Russia; savilov@mail.ru

**Keywords:** biopolymer, XRD, TGA, EDLC, DSSC

## Abstract

An ionic liquid (IL) 1-ethyl, 2-methyl imidazolium thiocyanate incorporated biopolymer system is reported in this communication for applications in dual energy devices, i.e., electric double-layer capacitors (EDLCs) and dye-sensitized solar cells (DSSCs). The solution caste method has been used to synthesize ionic-liquid-incorporated biopolymer electrolyte films. The IL mixed biopolymer electrolytes achieve high ionic conductivity up to the order of 10^−3^ S/cm with good thermal stability above 250 °C. Electrical, structural, and optical studies of these IL-doped biopolymer electrolyte films are presented in detail. The performance of EDLCs was evaluated using low-frequency electrochemical impedance spectroscopy, cyclic voltammetry, and constant current charge–discharge, while that of DSSCs was assessed using J–V characteristics. The EDLC cells exhibited a high specific capacitance of 200 F/gram, while DSSCs delivered 1.53% efficiency under sun conditions.

## 1. Introduction

Technologies are developed with the aim of serving humanity to make life easier and faster. Technologies have both positive and negative impacts on both living creatures and the lithosphere, hydrosphere, and atmosphere. Thousands of researchers are working day and night in the field of research and development to develop new technologies which can minimize the hazardous impacts of existing technologies, and also enhance their existing limitations [1,2,3,4,5]. The development of promising alternate bio-degradable electrochemical systems and their application in electrochemical devices could contribute to less polluting, environmentally friendly devices. Synthetic polymers are the main concern due to their adverse impacts on the ecosystem because of wide day-to-day applications as stabilizers, thickeners, binders, dispersants, lubricants, adhesives, carrier bags, etc. [5,6,7]. These polymers are non-biodegradable and endure for more than 20 years without decaying, which contributes to environmental pollution. Developing alternate bio-degradable polymers extracted from plants and animals, such as starch, protein, lipids, etc., to replace existing synthetic polymers such as polyether, polypropylene, polyethene, etc., could contribute to the development of environmentally friendly uses. Many research opportunities in the application of biopolymers in industries such as medical, food, cosmetics, and pharmaceutical are already in progress; however, our focus here was on the synthesis of highly conductive ionic-liquid-doped biopolymer electrolytes for application in highly efficient, stable, and reliable electrochemical devices [8,9,10].

Electrolytes are the backbone of any electrochemical devices containing electrodes, i.e., electrolyte systems such as batteries, fuel cells, super capacitors, and sensitizer-based solar cells. Electrolytes are generally ionic conductors with a conductivity between 10^−2^ and 10^−5^ S/cm. The transmission of charge within electrochemical cells is governed by the cations and anions present in the electrolyte, whereas the flow of charge in external circuits is governed by electrons to maintain electrical equilibrium within the cell [11,12]. Conventional electrolytes are generally diluted strong acid/base, 1–2 molar salt solutions and ionic liquids. These acid, base, and salt solutions provide sufficient cations and anion to the electrolyte to ensure proper functioning. These electrolytes are generally liquid in nature and are highly volatile, corrosive, and dry out over time, which degrades the performance of the device [13,14]. In order to tackle such maintenance issues, polymer electrolytes have been developed, the application of which will have various positive impacts on the performance of the devices. Compared with liquid electrolytes, polymer electrolytes have fewer drawbacks and many advantages [15]. Polymer electrolytes are generally gel, quasi-solid, or solid in nature. Various synthetic polymers, natural polymers, semi-synthetic polymers, and biopolymers have been doped with salts, weak acids/bases, and ionic liquids in various compositions to synthesize highly conductive, durable electrolytes to fulfil electrochemical requirements. Replacing the existing non-biodegradable synthetic polymers with biodegradable polymers will contribute to tackling environmental issues to some extent [16,17,18].

Conventional capacitors work through the electric polarization of dielectric molecules by aligning the dipole towards the direction of the applied electric field, whereas in the case of EDLCs, charge storage occurs by means of physical separation towards the respective electrodes; this provides a higher charge storage capacity, delivering higher capacitance. The mechanism involves the physical deposition of charge at the electrodes, hence the necessity of large-specific-area porous electrodes for EDLC [19,20,21]. Carbon material such as graphene, activated porous carbon, carbon nano tubes (CNTs), graphene oxide (GO), and reduced graphene oxide (rGO) are actively being used as electrode materials for supercapacitor [22]. Solar cells are mainly divided into two categories: silicon solar cells and electrolyte-based solar cells. Silicon solar cells are the best in any case due to their longer life span and high photon conversion efficiency. The main limitation of this type of solar cells is the manufacturing procedure, which is expensive and becomes unaffordable in lots of developing as well as underdeveloped countries [23,24,25,26]. A new class of solar cell has been developed based on organic and inorganic materials and compounds; these are much cheaper, the manufacturing process is much simpler, and they can be made with little infrastructure. DSSCs, quantum-dot-sensitized solar cell (QDSCs), and perovskite-sensitized solar cell (PSSC) are examples of such solar cells [27,28,29]. These solar cells are still in developmental and testing phases. The main challenges of these types of solar cells are the photon conversion efficiency and stability. Efficiency as high as 13% for DSSCs and more than 17% for PSSCs under laboratory conditions have been reported thus far [30,31].

Most electrochemical devices comprise electrodes and electrolytes. The essential condition of polymer electrolytes is high ionic conductivity. However, for polyether-based electrolytes, the most popular method is adding ionic salts. Adding ionic liquid (IL) is a novel approach which has appeared in the literature. In this study, a biopolymer doped with IL (1-ethyl, 2-methyl imidazolium thiocyanate) was used, replacing the aqueous phase electrolytes. The application of the optimized IL-doped biopolymer electrolyte (ILBPE) was employed in the fabrication of both EDLC and DSSC devices.

## 2. Results and Discussion

### 2.1. Ionic Conductivity

**Figure 1:**
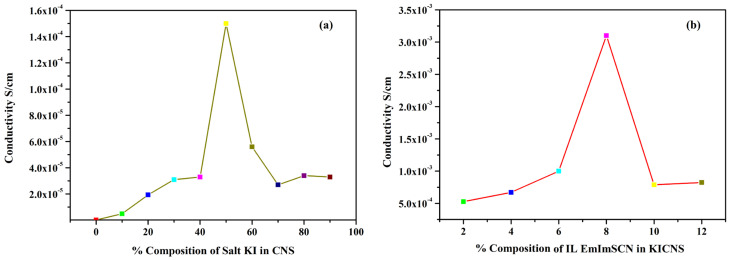
Ionic conductivity of (**a**) KICNS and (**b**) ILCNS.

To measure the bulk resistance of all the synthesized KIBPEs and ILBPEs, electrochemical impedance spectroscopy technique was used within a frequency range of 10 Hz to 10^6^ Hz. The ionic conductivity (σ) of the films was calculated using Equation (1).

(1) $$\sigma =\frac{L}{R_{b}A}$$

where *R_b_* is the bulk resistance, L is the thickness, and *A* represents the area of the electrode used to sandwich the films. The thickness of both the KIBPE and ILBPE films was between 0.014 cm and 0.018 cm. The overall conductivity values of all films, determined using EIS measurements, are tabulated in Table 1 and plotted in Figure 1. Thus, it is clear that for both the BPE and ILBPE systems, the conductivity increased initially, attained maxima, and then decreased. In KIBPEs, we have achieved the conductivity maxima at 50 weights % concentration of KI with a conductivity value of 1.50 × 10^−4^ S/cm (Figure 1a), whereas in ILBPEs, we achieved the highest conductivity at 8 weight % concentration of IL with a conductivity value of 3.10 × 10^−3^ S/cm (Figure 1b). Single maxima as well as double maxima in dispersed polymer electrolyte systems have already been cited in the literature, where conductivity enhancements are due to additional charge carriers; lower maxima indicate the formation of charge pair ions [32,33]. Maximum conducting ILBPEs were sandwiched between the electrodes to fabricate EDLCs and DSSCs. In ILBPEs, the extant studies are restricted to a 12 weight% IL concentration due to the non-availability of free-standing biopolymer electrolyte film.

### 2.2. Wagner’s DC Polarization

**Figure 2:**
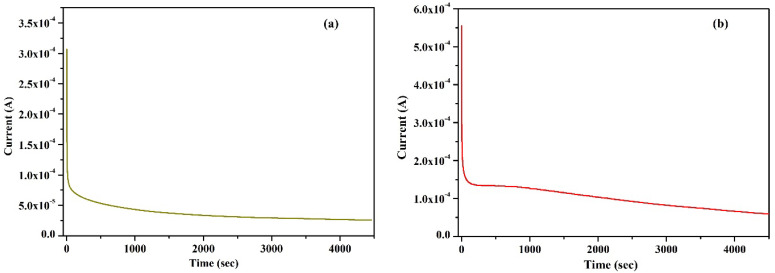
Wagner’s polarization of (**a**) KIBPE and (**b**) ILBPE polymer electrolyte films.

Wagner’s DC polarization method was applied to measure the ionic transference number of the highest conducting KIBPE and ILBPE films. A low DC voltage amplitude was applied across the film for 4500 s, as shown in Figure 2a,b. Initially, the current dramatically increased, then rapidly decreased over time, and finally stabilized. The stabilization of the current was due to the occurrence of electrode polarization upon the application of DC potential, which caused the deposition of ions across the electrode. The ionic transference number (*t_ion_*) was calculated using Equation (2) using the data evaluated from Figure 2.

(2) $$t_{ion}=\frac{initial\;cirrent-final\;current}{Initial\;current}$$

The calculated values of KIBPE and ILBPE were 0.97 (Figure 2a) and 0.94 (Figure 2b), respectively, which confirmed that both systems were predominantly ionic in nature.

### 2.3. Linear Sweep Voltammetry

**Figure 3:**
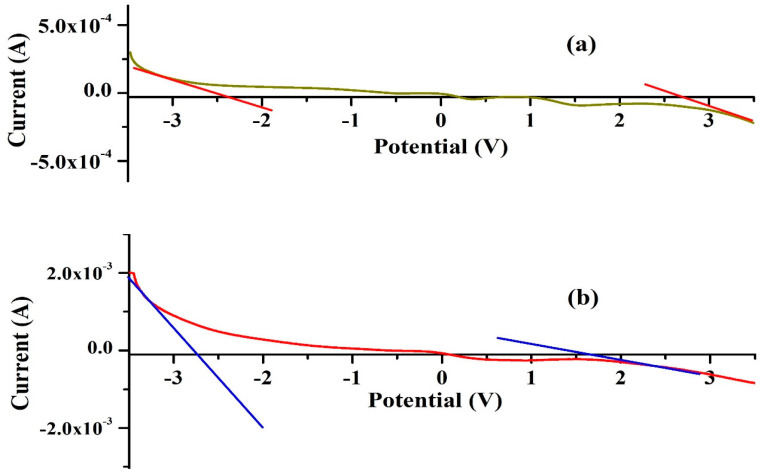
ESWs of (**a**) KIBPE and (**b**) ILBPE polymer electrolyte films.

The linear sweep voltammetry (LSV) technique was applied within a symmetrical voltage range of −3.5 V to 3.5 V to evaluate the electrochemical stability window (ESW) of the highest ionic conducting KIBPE and ILBPE films at a scan rate of 10 mV/s. Figure 3 shows the increase and decrease in the value of the current when the voltage ranged from −3.5 V to 3.5 V. For both the electrolyte films, it was observed that the value of the current decreased rapidly and tended to stabilize within their potential range. The region where the current tended to stabilize is known as the ESW of the films. ESWs of 5.16 volts (Figure 3a) and 4.3 volts (Figure 3b) were calculated for KIBPE and ILBPE, respectively. A high stability of 4.3 volt will definitely favor the fabrication of electrochemical devices.

### 2.4. X-ray Diffraction

**Figure 4:**
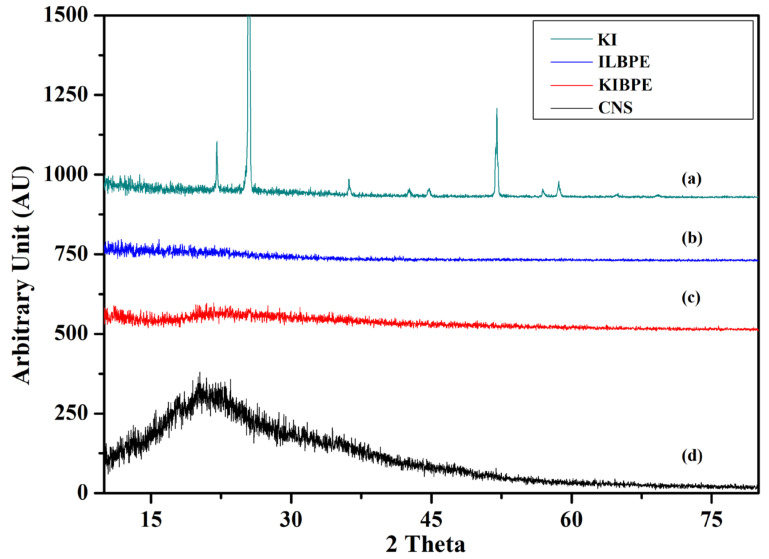
X-ray diffraction pattern of (**a**) KI, (**b**) ILBPE, (**c**) KIBPE, and (**d**) CNS.

Figure 4 shows the X-ray diffraction pattern of pure corn starch (CNS), KI, the highest conducting composition of the KIBPE film, and the highest conducting composition of the ILBPE films. The XRD pattern was recorded over a range of 2θ values from 10° to 80°. The XRD pattern of the KI salt (Figure 4a) exhibited a crystalline nature, with intense to less intense narrow peaks at 22°, 25°, 36°, 42°, 45°, 52°, 57°, and 58°, whereas the XRD pattern of CNS (Figure 4d) exhibited a broad peak at 20.5°, demonstrating the semi-crystalline nature of the CNS film. It is clear in Figure 4b,c that no peaks related to KI were detected for the KIBPE and ILBPE films, which indicates the complete dissolution of salt and IL into the matrix of the biopolymer. Additionally, a comparative study between KIBPE and ILBPE (Figure 4b,c) showed their broadness and hollow properties. Reductions in peak intensity, in addition to broadness and hollow characteristics, are clear indications of a suppressed crystallinity or enhanced amorphous nature achieved by doping salt/IL. It has been cited in the literature that the amorphous region provides more mobility with rich conductivity. These initial observations are supported by our conductivity, XRD, and POM studies [34].

### 2.5. FTIR Spectroscopy

**Figure 5:**
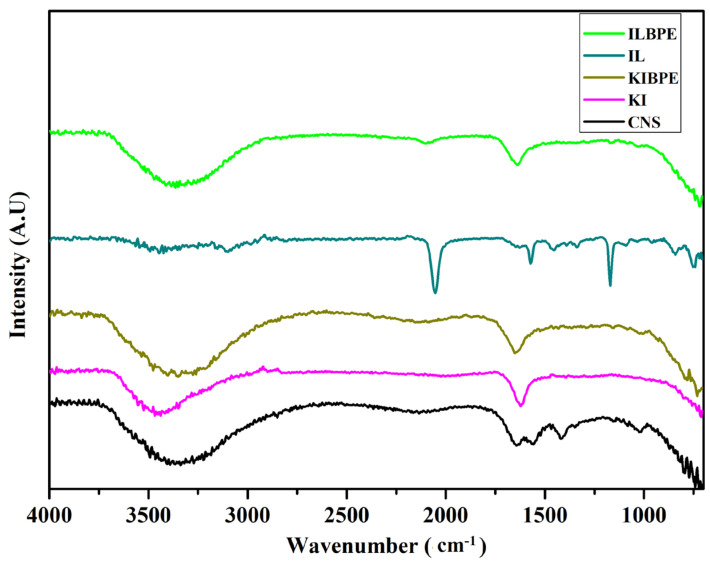
FTIR spectra of CNS, KI, IL, KIBPEs, and ILBPEs.

Figure 5 shows the FTIR spectra of the CNS, KI, and ionic liquid, the highest conducting composition of KIBPEs, and the highest conducting composition of ILBPEs.

Spectra recorded over a wavenumber range from 4000 cm^−1^ to 400 cm^−1^ are shown in Figure 5; their functional groups are tabulated in Table 2. It is clear that there is peak position shift in KIBPEs and ILBPEs, which demonstrates the complex nature of the films (i.e., the interactions between the biopolymer and salt/IL). Comparative studies between host and dispersoids clearly affirmed that ILBPEs did not contain any new peaks other than those of the host material, confirming the composite nature of ILBPE films.

### 2.6. Polarized Optical Microscopy

**Figure 6:**
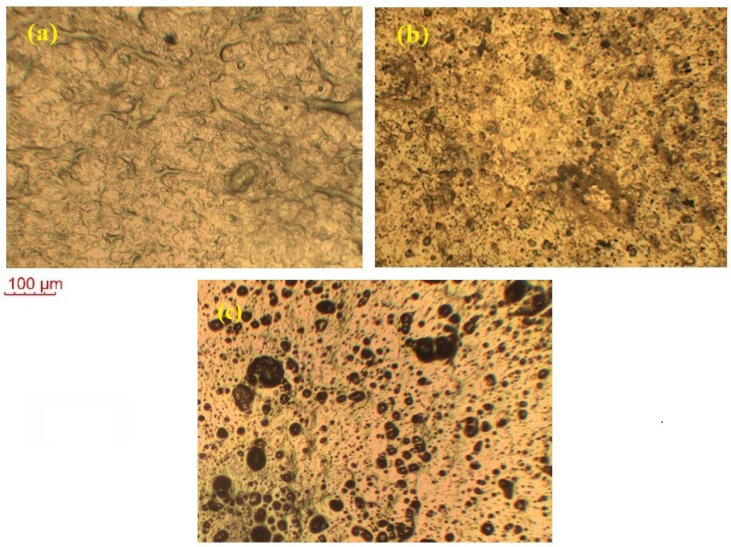
POM images of (**a**) pure CNS, (**b**) KIBPE polymer electrolyte film, and (**c**) ILBPE polymer electrolyte film.

Figure 6 shows the POM images of the pure CNS, KIBPE, and ILBPE films. The image of CNS in the figure depicts some distinguishably brighter patches, in addition to shadowy patches. The brighter patches imply the distribution of crystalline (bright) region and amorphous (shadowy) regions, as cited in the literature. Close comparative study of CNS, KIBPEs, and ILBPEs (Figure 6a–c) showed that adding IL/salt increased the distribution of the amorphous shadowy region as compared with the crystalline bright region, which is well supported by our conductivity and XRD data.

### 2.7. Thermogravimetric Analysis

**Figure 7:**
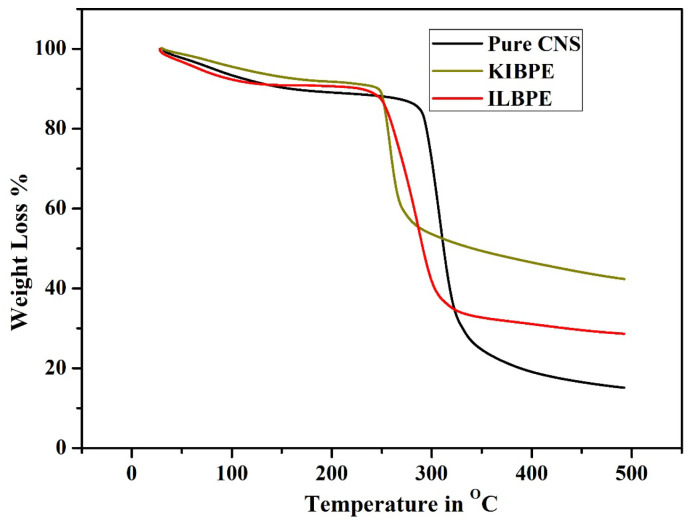
Thermogravimetric analysis of the synthesized films.

Figure 7 shows the thermogravimetric analysis (TGA) results of pure CNS, KIBPE film, and ILBPE film; the TGA was performed within a temperature range from 30 °C (room temperature) to 500 °C (high temperature). From Figure 7, it is evident that all the three different films suffered initial weight loss up to 100 °C, which is due to the presence of traces of manure in DD water within the films. The pure CNS film was stable up to a temperature of 280 °C; KIBPE and ILBPE films were stable up to a temperature of 250 °C. Weight loss at higher temperatures is due to pre-carbonization and high-temperature reactions. The higher temperature stability of the KIBPE and ILBPE films indicates their suitability for application in devices at normal operating temperature, as well as extreme temperature conditions.

## 3. Device Application and Performance

### 3.1. Fabrication of the EDLC

Graphite sheets 1 cm^2^ in size were cut using a paper cutter and used as current collector electrodes. Porous-activated carbon synthesized from waste plastic with a surface area greater than 800 m^2^/g was used as the active material. To coat the active material onto the surface of the current collector, a slurry containing 10% PVDF (as binder) and 100 mg of active material were thoroughly mixed using an agate mortar and pestle in the presence of an acetone solvent. The homogeneous slurry was coated using a paintbrush and dried at 100 °C; once dried, the electrodes were then stored before use as EDLC electrodes. The highest conducting polymer electrolyte was sandwiched between two electrodes to fabricate the EDLC.

### 3.2. Fabrication of the DSSC

Fluorine-doped tin oxide (FTO) conducting glass sheet was cut into sizes of 3 cm^2^ and washed several times with a solution of DD water and acetone at a ratio of 50:50 in an ultrasonic bath and used as current collector electrodes for both the working and counter electrodes of the DSSC. Initially, a thin layer of dilute titanium diisopropoxide bis(acetylacetonate) was coated over the FTO glass using a spin coater, then annealed at 500 °C for 30 min, which enabled blocking. Above the blocking layer, a thick layer of nano-porous titania paste was coated and further annealed at 500 °C for 30 min to remove any impurities and to achieve a thickness of 10 microns. The resultant electrode was finally dipped in a N_7_ dye solution at a concentration of 0.5 millimolars for 6–8 h. The electrode was removed from the dye solution and washed with ethanol to remove the loosely bound dye molecule, then dried at 50 °C and stored before use as the working electrode of the DSSC. Another clean and dried FTO sample was coated with a diluted solution of chloroplatinic acid using a spin coater and annealed at 500 °C for 30 min, then stored for use as the counter electrode of the DSSC. The highest conducting ILBPE was doped with 10% I_2_ with respect to KI for redox couple formation, and then sandwiched between the working and the counter electrodes to fabricate the DSSC.

### 3.3. Performance of the EDLC

#### 3.3.1. Low-Frequency Electrochemical Impedance Spectroscopy

**Figure 8:**
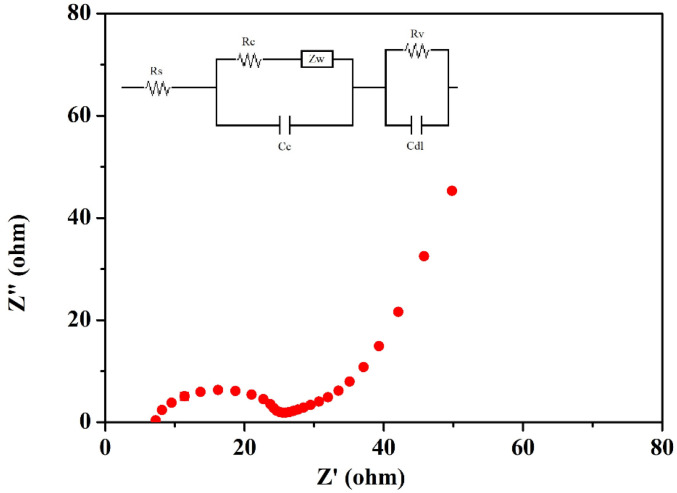
LFEIS of the fabricated EDLC.

LFEIS analysis of the EDLC cell fabricated using the highest conducting ILBPE was carried out within the low-frequency range. The Nyquist plot is shown in Figure 8. It is clearly seen that the spectra are divided into three different regions. A semi-circular section towards the high-frequency region showed its purely dielectric nature; the linear region making an angle of 40°–45° with the Z′ at mid-frequencies indicated the electrode–electrolyte interaction; and the low-frequency region making an angle greater than 45° with Z′ indicated electrode polarization, where the ions were absorbed towards the porous electrode. The probable equivalent circuit of the Nyquist plot was also fitted and analyzed using fitting software available in the electrochemical work station CHI 610D with the CH instrument, as depicted in the inset image of Figure 8. The values of different components, R_s_, R_c_, R_v_, Z_w_, C_c_, and C_dl_, were obtained as 6.61 Ω, 10.63 Ω, 7.83 Ω, 0.056 Ω, 3.33 × 10^−5^ F, and 4.81 × 10^−7^ F, respectively. The specific capacitance, *C_sp_*, of the cell was also calculated using Equation (3), where *υ* is the applied low frequency, *Z* is the complex part of the impedance, and m is the mass of active materials loaded on each electrode. A specific capacitance as high as 176 F/g was obtained from the fabricated EDLCs at a frequency of 10^−2^ Hz.

(3) $$C_{sp}=\frac{m}{2\pi \upsilon Z^{\prime \prime }}$$

#### 3.3.2. Cyclic Voltammetry

**Figure 9:**
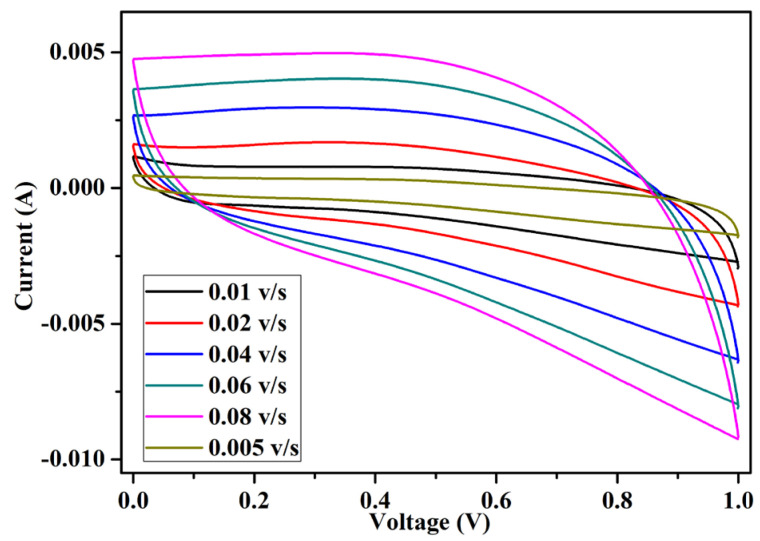
Cyclic voltammetry of the fabricated EDLC.

The cyclic voltammetry (CV) technique was performed for the fabricated EDLC to study the charge storage mechanism. In this technique, a linear sweeping voltage varying between 0 and 1 V was applied across the EDLC cells at scan rates of 0.005 v/s to 0.08 v/s (Table 3). The cyclic voltammetry graph is plotted in Figure 9. The cyclic voltammogram plots are quite rectangular in shape, with some inclination toward the y-axis (current). The inclination was identified in the EDLC while sweeping from 0 to 1 volts and 1 to 0 volts. The inclination region was due to the charging and discharging of the capacitor. The perfect rectangular CV shape was only achieved using conventional dielectric capacitors. The specific capacitance values (*C_sp_*) at different scan rates of the EDLC cell were also calculated using Equation (4), where A is the average area under the curve, *V* is the voltage window, and *S* is the scan rate. The specific capacitance values were calculated, as detailed in Table 3.

(4) $$C_{sp}=\frac{A}{mVS}$$

#### 3.3.3. Constant Current Charging and Discharging

**Figure 10:**
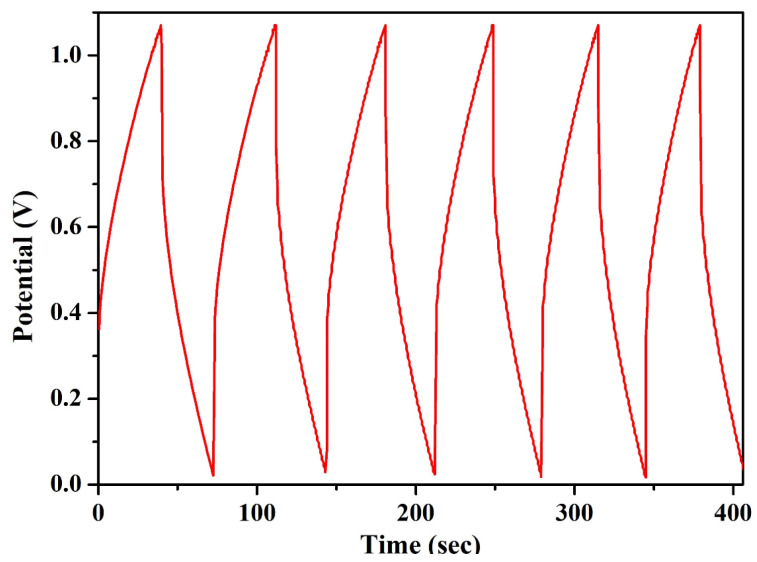
CCD of the EDLC fabricated in our own laboratory.

Constant current charging and discharging (CCD) was carried out for the EDLC at a constant current of 4 mA and in a relative charging and discharging potential window of 1.06 volt. The first five cycles of all EDLC cells are plotted in Figure 10. The charge–discharge curves of all EDLC cells contained a linear region, varying with time, for both the charging and discharging cycles. Various necessary parameters of the EDLC cell, such as discharge specific capacitance (C*_dsp_*), coulombic efficiency (C*_ef_*), specific energy density (E*_d_*), and specific power density (P*_d_*) were calculated using Equations (5)–(8), where I is the applied constant current for charging and discharging, D*_t_* is the discharge time, *V* is the relative discharge potential, and C*_t_* is the charging time. The EDLC cell could deliver a specific discharge capacitance of as high as 200 F/g, a coulombic efficiency higher than 80%, an energy density as high as 35 Wh/kg, and a power density of 4080 W/kg. The EDLC delivered promising results, which proves its potential over existing electrolytes.

(5) $$C_{dsp}=\frac{ID_{t}}{mV}$$

(6) $$C_{ef}=\frac{C_{t}}{D_{t}}$$

(7) $$E_{d}=\frac{C_{dsp}V^{2}}{2*3.6}$$

(8) $$P_{d}=\frac{E_{d}\times 3600}{D_{t}}$$

### 3.4. Performance of the DSSC

**Figure 11:**
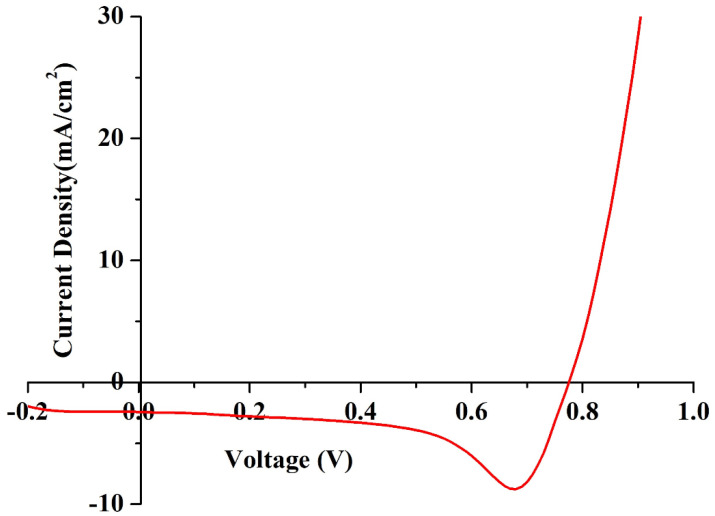
J–V characteristics of the fabricated DSSC under one sun conditions.

The J–V characteristics of DSSCs were recorded using a solar simulator to measure the overall performance and charge transfer mechanism. In this technique, a sweeping voltage is applied across the working and counter electrodes of the DSSC, irradiated at a condition of one sun (1000 W/m^2^) and at a scan rate of 0.1 v/s. The value of the current density with respect to voltage was recorded, and the J–V curve was plotted, as shown in Figure 11. Key parameters of fill factor (FF) and photon conversion efficiency (η) were calculated using the formulae presented in Equations (9) and (10), respectively, where V*_oc_* is the open circuit voltage, J*_sc_* is the short circuit current, V*_max_* is the peak voltage, and J*_max_* is the peak load current. The DSSC was able to deliver an η of 1.53% with an FF of 72. The DSSC also generated V*_oc_*, J*_sc_*, V*_max_*, and J*_max_* values of 0.77 V, 2.43 mA/cm^2^, 0.76 V, and 2.02 mA/cm^2^, respectively.

(9) $$FF=\frac{V_{max}\times J_{max}}{V_{oc}\times J_{sc}}$$

(10) $$\eta =\frac{V_{oc}\times J_{sc}\times FF}{p_{in}}$$

## 4. Materials and Method

### 4.1. Materials Used

Corn starch (CNS), potassium iodide (KI), polyvinylidene fluoride (PVDF), and acetone were purchased from Hi-Media, India; the ionic liquid (IL), 1-ethyl-3-methylimidazolium thiocyanate (EmImSCN), was purchased from TCI Chemical India Private Limited, India; fluorine-doped tin oxide (FTO), titania paste, N_7_ dye, and titanium diisopropoxide bis(acetylacetonate) were purchased from Sigma Aldrich, India; graphite sheets were purchase from Nikunj Pvt. Ltd. Mumbai, India; DD water was produced within the laboratory using a distillation unit.

### 4.2. Synthesis of Biopolymer Electrolyte

The corn starch doped with ionic liquid (EmImSCN) and KI biopolymer electrolytes were synthesized using the solution caste technique. Following a common procedure, initially, the host polymer was fixed at 300 mg, then dissolved in DD water, after which 30 mg of glutaraldehyde was added as a preservative agent to avoid any bacterial and fungal growth. Multiple samples of CNS and glutaraldehyde were prepared at 50 °C, to which different weight % concentrations (10–90) of KI were added, then stirred for 6 h until homogenous, uniform mixtures were formed. The synthesized KI mixed biopolymer electrolytes (KIBPEs) were poured into the polypropylene Petri dish and allowed to dry overnight at 50 °C. Different ionic EmImSCN liquids of varying weight% concentrations (2, 4, 6, 8, 10, 12, 14, 16, and 18) were added to multiple solutions of the highest conducting KI-doped biopolymer electrolyte and stirred overnight to obtain homogenous mixtures of ionic-liquid-doped biopolymer electrolyte solutions. The homogenous solutions were poured into polypropylene Petri dishes and dried overnight at 50 °C, followed by vacuum dying to evaporate any minute traces of solvent if present. Finally, various characterizations were used for various analyses; the results are presented in detail.

## 5. Characterization Techniques

### 5.1. Electrolytes

For the KIBPEs and ILBPEs, electrochemical impedance spectroscopy (EIS) was used to measure the bulk resistance of all the synthesized KIBPE and ILBPE films to determine the highest conducting compositions. Wagner’s DC polarization was used to calculate the ionic transference number (t_ion_); linear sweep voltammetry was used to identify the electrochemical stability window (ESW). X-ray diffraction (XRD) and polarized optical microscopy (POM) were used for structural analysis, followed by Fourier-transform infrared spectroscopy (FTIR) to study interactions among the materials; thermogravimetric analysis (TGA) was performed to measure the thermal stability.

### 5.2. Devices

For the electric double layer capacitor (EDLC), low-frequency electrochemical impedance spectroscopy (LFEIS) and cyclic voltammetry (CV) were used to measure the specific capacitance and constant current charging and discharging (CCD), respectively, identifying the specific capacitance, coulombic efficiency, power density, and energy density, which are some key parameters related to the performance. J–V characteristics were recorded using a solar simulator to measure the overall photon conversion efficiency (η) of the DSSC. EIS measurements of the fabricated DSSC were carried out to determine the charge transfer mechanism of the DSSC.

## 6. Conclusions

Highly conductive ionic-liquid-incorporated biopolymer electrolytes seem to be good candidates for applications in electrochemical devices. Electrochemical impedance spectroscopy (EIS) showed that conductivity increased with IL doping and a value as high as 10^−3^ S/cm was achieved, which is acceptable for application in electrochemical devices. This conductivity enhancement is attributed to the increase in the number of charge carriers or the reduction in crystallinity by IL incorporation. XRD and POM studies further confirmed the reduction in crystallinity (more amorphous nature) in the more conductive region. The complexation and composite nature were further confirmed by FTIR spectroscopy, while the thermal stability results (TGA) confirmed that our biopolymer electrolyte was stable to a temperature of 280 °C. The fabricated sandwich EDLC and DSSC devices demonstrated remarkable performance, with capacitance values as high as 200 F/g; the DSSC exhibited an efficiency of 1.53% under one sun conditions.

## Figures and Tables

**Table 1:** Ionic conductivity of KICNS and ILCNS.

KICNS		ILCNS	
Composition	Conductivity S/cm	Composition	Conductivity S/cm
0	2.30 × 10^−7^	2	5.26 × 10^−4^
10	4.90 × 10^−6^	4	6.71 × 10^−4^
20	1.94 × 10^−5^	6	1.00 × 10^−3^
30	3.10 × 10^−5^	8	3.10 × 10^−3^
40	3.30 × 10^−5^	10	7.88 × 10^−4^
50	1.50 × 10^−4^	12	8.24 × 10^−4^
60	5.60 × 10^−5^		
70	2.70 × 10^−5^		
80	3.40 × 10^−5^		
90	3.30 × 10^−5^		

**Table 2:** FTIR spectra of host material and their complexes.

Host	FTIR Spectra	Functional Group
EmIm^+^	966–1093 cm^−1^	C=H bending
	1174 cm^−1^	C-H stretching
	1337 cm^−1^	O-H bending
	1460–1440 cm^−1^	C-H bending
	1563 cm^−1^	N-O stretching
	3000–2840 cm^−1^	Thiocynate
SCN^−^	2042 cm^−1^	Thiocynate
KI	1625 cm^−1^	C=C stretching
Corn starch	3200–3500 cm^−1^	-OH bond
	3000–2840 cm^−1^	C-H alkane
	1640 cm^−1^	C=H alkene
	1553 cm^−1^	N-O stretching
	1400–1300 cm^−1^	HC-OH bending
	1151 cm^−1^	S=O stretching
	1076 cm^−1^	C-O stretching
KIBPE	1647 cm^−1^	C=C stretching
ILBPE	2087 cm^−1^	Thiocynate

EmIm^+^ and SCN^−^ are cation and anions of the IL EmImSCN.

**Table 3:** Specific capacitance of the EDLC cell at different scan rates.

Scan Rate v/s	Specific Capacitance F/g
0.005	200
0.01	179
0.02	146
0.04	120
0.06	105
0.08	95

## Data Availability

Data are available from the authors upon reasonable request.

## Sample Availability

Samples of the compounds are available upon reasonable request.

## References

[1] Singh R., Gautam S., Sharma B., Jain P., Chauhan K.D. (2021). Biopolymers and their classifications. Biopolymers and Their Industrial Applications.

[2] Tabasum S., Younas M., Zaeem M.A., Majeed I., Majeed M., Noreen A., Iqbal M.N., Zia K.M. (2019). A review on blending of corn starch with natural and synthetic polymers, and inorganic nanoparticles with mathematical modeling. Int. J. Biol. Macromol..

[3] Chong M.Y., Numan A., Liew C.-W., Ramesh K., Ramesh S. (2017). Comparison of the performance of copper oxide and yttrium oxide nanoparticle based hydroxylethyl cellulose electrolytes for supercapacitors. J. Appl. Polym. Sci..

[4] Liu S., Liu W., Ba D., Zhao Y., Ye Y., Li Y., Liu J. (2023). Filler-integrated composite polymer electrolyte for solid-state lithium batteries. Adv. Mater..

[5] Wu Y., Li Y., Wang Y., Liu Q., Chen Q., Chen M. (2022). Advances and prospects of PVDF based polymer electrolytes. J. Energy Chem..

[6] Ge X., Zhang F., Wu L., Yang Z., Xu T. (2022). Current Challenges and Perspectives of Polymer Electrolyte Membranes. Macromolecules.

[7] Monisha S., Mathavan T., Selvasekarapandian S., Benial A.M.F., Aristatil G., Mani N., Premalatha M., Pandi D.V. (2017). Investigation of bio polymer electrolyte based on cellulose acetate-ammonium nitrate for potential use in electrochemical devices. Carbohydr. Polym..

[8] Rayung M., Aung M.M., Azhar S.C., Abdullah L.C., Su’ait M.S., Ahmad A., Jamil S.N.A.M. (2020). Bio-Based Polymer Electrolytes for Electrochemical Devices: Insight into the Ionic Conductivity Performance. Materials.

[9] Rahman N.A., Abu Hanifah S., Mobarak N.N., Ahmad A., Ludin N.A., Bella F., Su’Ait M.S. (2021). Chitosan as a paradigm for biopolymer electrolytes in solid-state dye-sensitised solar cells. Polymer.

[10] Jothi M.A., Vanitha D., Sundaramahalingam K., Nallamuthu N. (2022). Utilisation of corn starch in production of ‘eco friendly’ polymer electrolytes for proton battery applications. Int. J. Hydrog. Energy.

[11] Chavez-Esquivel G., García-Martínez J.C., Cervantes-Cuevas H., Acosta D., Vera-Ramírez M.A. (2022). Effect of thermo-alkali treatment on the morphological and electrochemical properties of biopolymer electrolytes based on corn starch–Al(OH)_3_. Polym. Bull..

[12] Abdulwahid R.T., Aziz S.B., Kadir M.F. (2022). Design of proton conducting solid biopolymer blend electrolytes based on chitosan-potato starch biopolymers: Deep approaches to structural and ion relaxation dynamics of H^+^ ion. J. Appl. Polym. Sci..

[13] Jothi M.A., Vanitha D., Bahadur S.A., Nallamuthu N. (2021). Promising biodegradable polymer blend electrolytes based on cornstarch: PVP for electrochemical cell applications. Bull. Mater. Sci..

[14] Shi J., Shi B. (2021). Environment-Friendly Design of Lithium Batteries Starting from Biopolymer-Based Electrolyte. Nano.

[15] Jothi M.A., Vanitha D., Nallamuthu N., Manikandan A., Bahadur S.A. (2020). Investigations of lithium ion conducting polymer blend electrolytes using biodegradable cornstarch and PVP. Phys. B Condens. Matter.

[16] Rayung M., Aung M., Ahmad A., Su’Ait M., Abdullah L.C., Jamil S.A.M. (2019). Characteristics of ionically conducting jatropha oil-based polyurethane acrylate gel electrolyte doped with potassium iodide. Mater. Chem. Phys..

[17] Singh R., Singh P.K., Singh V., Bhattacharya B. (2019). Quantitative analysis of ion transport mechanism in biopolymer electrolyte. Opt. Laser Technol..

[18] Kumar L.S., Selvin P.C., Selvasekarapandian S. (2021). Impact of lithium triflate (LiCF_3_SO_3_) salt on tamarind seed polysaccharide-based natural solid polymer electrolyte for application in electrochemical device. Polym. Bull..

[19] Ahuja H., Dhapola P.S., Rahul, Sahoo N.G., Singh V., Singh P.K. (2020). Ionic liquid (1-hexyl-3-methylimidazolium iodide)-incorporated biopolymer electrolyte for efficient supercapacitor. High Perform. Polym..

[20] Wang J., Liang Y., Zhang Z., Ye C., Chen Y., Wei P., Wang Y., Xia Y. (2021). Thermoplastic starch plasticized by polymeric ionic liquid. Eur. Polym. J..

[21] Devi L.S., Das A.B. (2020). Effect of ionic liquid on sol-gel phase transition, kinetics and rheological properties of high amylose starch. Int. J. Biol. Macromol..

[22] Mohit, Yadav N., Hashmi S. (2022). High energy density solid-state supercapacitors based on porous carbon electrodes derived from pre-treated bio-waste precursor sugarcane bagasse. J. Energy Storage.

[23] Rosli N.A.H., Loh K.S., Wong W.Y., Yunus R.M., Lee T.K., Ahmad A., Chong S.T. (2020). Review of Chitosan-Based Polymers as Proton Exchange Membranes and Roles of Chitosan-Supported Ionic Liquids. Int. J. Mol. Sci..

[24] Ren F., Wang J., Xie F., Zan K., Wang S., Wang S. (2020). Applications of ionic liquids in starch chemistry: A review. Green Chem..

[25] Salama A., Hesemann P. (2020). Recent Trends in Elaboration, Processing, and Derivatization of Cellulosic Materials Using Ionic Liquids. ACS Sustain. Chem. Eng..

[26] Chen P., Xie F., Tang F., McNally T. (2020). Ionic Liquid (1-ethyl-3-methylimidazolium acetate) Plasticization of Chitosan-Based Bionanocomposites. ACS Omega.

[27] Alday P.P., Barros S.C., Alves R., Esperança J.M., Navarro-Segarra M., Sabaté N., Silva M.M., Esquivel J.P. (2020). Biopolymer electrolyte membranes (BioPEMs) for sustainable primary redox batteries. Adv. Sustain. Syst..

[28] Sun Z., Yang L., Zhang D., Song W. (2019). High performance, flexible and renewable nano-biocomposite artificial muscle based on mesoporous cellulose/ ionic liquid electrolyte membrane. Sens. Actuators B Chem..

[29] Torres F.G., De-La-Torre G.E. (2021). Algal-based polysaccharides as polymer electrolytes in modern electrochemical energy conversion and storage systems: A review. Carbohydr. Polym. Technol. Appl..

[30] Venkatesan S., Lin W.-H., Teng H., Lee Y.-L. (2019). High-Efficiency Bifacial Dye-Sensitized Solar Cells for Application under Indoor Light Conditions. ACS Appl. Mater. Interfaces.

[31] Dai T., Cao Q., Yang L., Aldamasy M.H., Li M., Liang Q., Lu H., Dong Y., Yang Y. (2021). Strategies for High-Performance Large-Area Perovskite Solar Cells toward Commercialization. Crystals.

[32] Hoang H.M., Van Pham T.B., Grampp G., Kattnig D.R. (2014). Exciplexes versus Loose Ion Pairs: How Does the Driving Force Impact the Initial Product Ratio of Photoinduced Charge Separation Reactions?. J. Phys. Chem. Lett..

[33] Lakshmi N., Chandra S. (2002). Ion transport in some solid state proton conducting composites studied from volta cell e.m.f. and complex impedance spectroscopy. Bull. Mater. Sci..

[34] Sharma T., Gultekin B., Dhapola P.S., Sahoo N., Kumar S., Agarwal D., Jun H., Singh D., Nath G., Singh P.K. (2022). Ionic liquid doped Poly (methyl methacrylate) for energy applications. J. Mol. Liq..

